# Ultrasound-guided pulsed radiofrequency for neuromyelitis optica spectrum disorder-associated neuropathic pain: A case report

**DOI:** 10.1097/MD.0000000000032417

**Published:** 2022-12-23

**Authors:** Fei Yang, Liheng Lin, Yu Xia, Changxue Wu

**Affiliations:** a Department of Pain, The Affiliated Hospital of Southwest Medical University, Luzhou, China; b Department of Anesthesiology, The Affiliated Hospital of Southwest Medical University, Luzhou, China.

**Keywords:** neuromyelitis optica spectrum disorder, neuropathic pain, pulsed radiofrequency

## Abstract

Neuropathic pain (NP) is one of the most prevalent and troublesome symptoms of neuromyelitis optica spectrum disorder (NMOSD), seriously affecting the patient’s life. At present, effective treatment for NP induced by NMOSD does not exist. Pulsed radiofrequency (PRF), an emerging microinvasive therapy, alleviates pain and is widely used to treat various types of NP. This is the first report describing a patient with NMOSD-associated NP treated with PRF on the left cervical 6 nerve root.

**Methods:**

A 49-year-old female with NMOSD-associated severe NP in the left upper limb and left shoulder tried several medications, but none were effective. She was diagnosed with NP caused by NMOSD.To alleviate severe pain, we performed PRF on the left cervical nerve root under the guidance of ultrasound. This treatment was repeated 3 times.

**Results:**

The patient’s pain was significantly relieved, with a visual analog scale score decreasing from 7-8/10 to 2-3/10, which was maintained during the 3-month follow-up period, without complications.

**Conclusion:**

PRF might be effective for the management of intractable neuropathic pain caused by NMOSD.

## 1. Introduction

Neuromyelitis optica spectrum disorder (NMSOD) is an autoimmune disease of the central nervous system that causes multiple severe neurological deficits.^[1–3]^ Neuropathic pain (NP), a common symptom of NMSOD, is often severe and intractable,^[4–6]^ affecting more than 85% of patients,^[5,7]^ and often resulting in a significant burden on the physical and mental health of patients, thus seriously reducing quality of life.^[6,8–10]^ At present, there is no standardized effective treatment for central NP caused by NMOSD. The main treatment is pharmacotherapy, including antiepileptics, antidepressants, and nonsteroidal anti-inflammatory drugs^[11,12]^; however, most patients respond poorly to drugs.^[5,7,8,13,14]^ Therefore, there is an urgent need to develop innovative techniques with better therapeutic efficacy for NMOSD patients with NP.

Pulsed radiofrequency (PRF) is an emerging, novel technology for alleviating pain. In PRF, a low-energy electrical field is delivered in rapid pulsations to the target nerve to block pain conduction pathways without damaging the nerve.^[15,16]^ PRF is regarded as a safe and effective treatment for NP, specifically when other treatments do not relieve pain.^[17]^

Here, we present the first case of NP after NMOSD that was successfully treated with PRF application to the cervical nerve roots.

## 2. Case presentation

**Figure 1.**
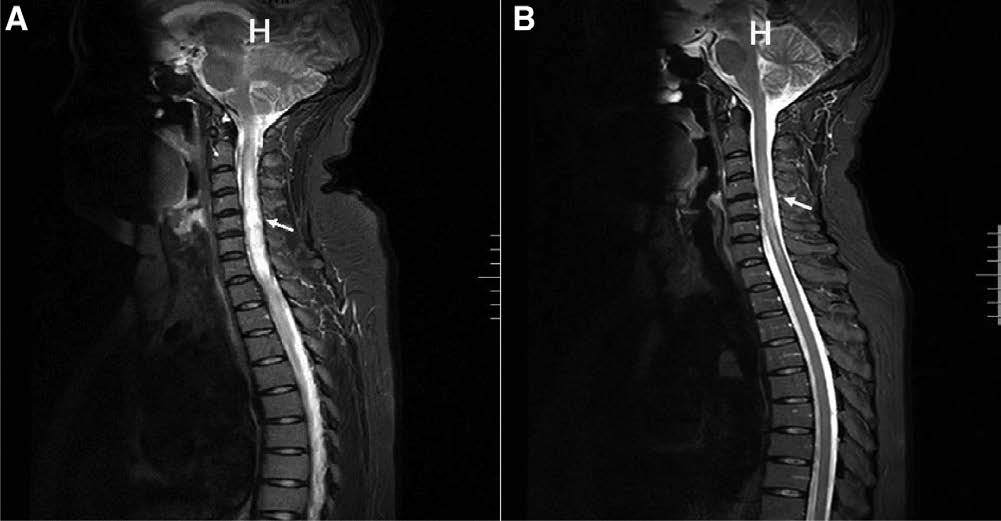
(A) Spine MRI on onset showed long strips and abnormal sheet signals from C2 to T3 with spinal cord swelling on T2WI (arrow). (B) Spine MRI on this admission showed abnormal signals from C3 to C7 and nearly complete resolution of spinal cord swelling on T2WI (arrow). H indicates head. MRI = magnetic resonance imaging, T1WI = T1-weighted images, T2WI = T2-weighted images.

A 49-year-old female presented to our neurology department with a sudden onset of left-sided extremity weakness 21 months ago. Neurological examination showed hypesthesia from the C7 plane and weakness on the left extremities. AQP4-IgG was positive in cerebrospinal fluid. Sagittal T2 imaging of the spine showed a high signal lesion on the spinal cord extending from C2 to T3, accompanied by cord swelling (Fig. 1A). She was diagnosed with NMOSD and underwent 14 days of high-dose glucocorticoid therapy and 5 days of gamma globulin shock therapy, with modest improvement in strength and no resolution of numbness. After discharge, she was started on prednisone and mycophenolate mofetil to prevent recurrence and was followed up regularly at the neurology department.

Approximately 3 months after the attack, she suffered from paroxysmal stabbing pain in her left upper limb and shoulder with occasional burning sensations. She went to the neurology department and was diagnosed with NMOSD-associated NP. She tried multiple medications, including pregabalin, gabapentin, duloxetine and tramadol. She reported that the pain was initially controlled with medication but the effectiveness of the treatment decreased as the drug dosage increased. She tried physiotherapy, but it was also ineffective. Four months ago, her pain gradually worsened and became so severe and constant that it affected her daily life and sleep. Thus, she was referred to our pain department. She complained of persistent stabbing pain in the left upper limb and left shoulder, with a burning sensation. The pain score was 7 to 8/10 (visual analog scale: 0 being no pain, 10 being unbearable pain). Neurological examination revealed 5-/5 distal muscle strength in the left upper limb according to the Medical Research Council scale, and hypesthesia in the left thumb. The remainder of the neurological examination was normal. Her signs and symptoms did not suggest cervical radiculopathy. Spinal magnetic resonance imaging showed decreased size and intensity of the lesion, and no new lesions were present (Fig. 1B). As she responded poorly to the pharmacological agents, she consented to PRF under ultrasound guidance to relieve her pain.

**Figure 2.**
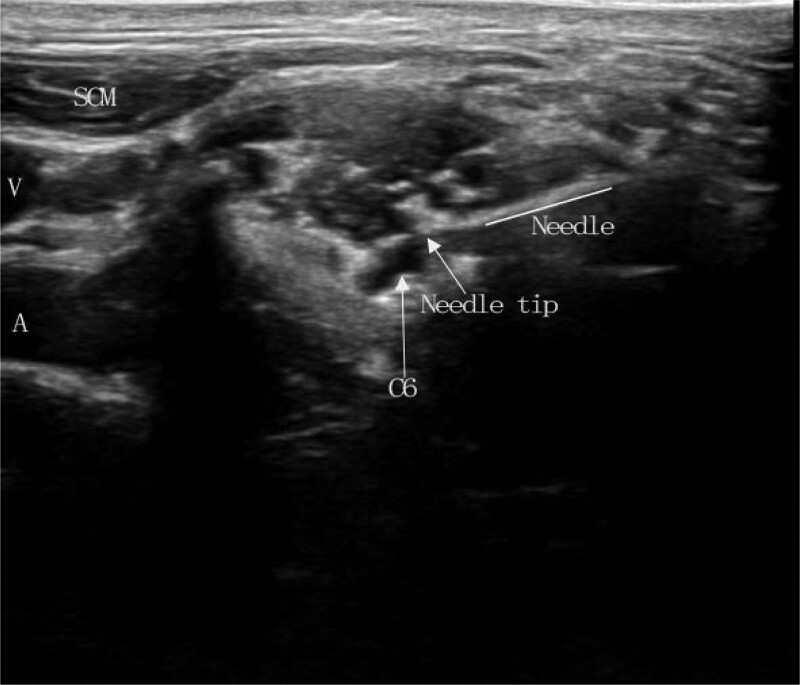
Ultrasound-guided pulsed radiofrequency of the left cervical 6 nerve root. C6 = the left cervical 6 nerve root, SCM = sternocleidomastoid muscle, V = jugular venous, A = carotid artery.

Because most of her pain was primarily located at C6, we opted to operate on the left C6 root. After disinfection and local anesthesia, a catheter needle (22 G, 10 cm, 5 mm) was injected near the left C6 nerve root under ultrasound guidance (Fig. 2), followed by sensory stimulation at 50 Hz, eliciting mild paresthesia over the painful regions. After the position had been determined, the PRF device was linked for the procedure (parameters: needle tip temperature: 42°C, single duration: 360 seconds, frequency: 2 Hz, pulse width: 20 ms). After her first treatment, her pain was partly relieved, so we repeated the procedure twice the following week. After 3 treatments, her pain was significantly relieved, with the visual analog scale score decreasing from 7-8/10 to 2-3/10. At the 3-month follow-up, she reported that pain relief was sustained, without any complications. Her sleeping issues were also resolved.

## 3. Discussion

Herein, we report on the first case of intractable NMOSD-associated NP treated with PRF, suggesting that PRF might be a potential treatment for NMOSD-associated NP.

NP is one of the most prevalent symptoms and one of the most significant influencers of quality of life in patients with NMOSD,^[10,18]^ which manifests primarily as persistent or intermittent sensations of “burning” and “tingling, pins and needles,” often located in the trunk, legs or arms.^[5,6,8,13,19]^ A recent cross-sectional cohort study of 166 NMOSD patients reported that 2/3 (64.2%) of patients receiving symptomatic treatment still had moderate to severe pain.^[8]^ Its mechanisms are not fully understood, no standard treatment has been established, and it remains a deeply challenging clinical condition. As mentioned above, the first-line treatment is pharmacotherapy. Unfortunately, as in the patient we reported, medication is often ineffective and has many side effects, such as dizziness, drowsiness, nausea, somnolence, and liver injury.^[1,8]^ Nonpharmacological treatments for NMOSD-associated NP are being explored. Recently, a randomized controlled study showed that scrambler therapy can significantly alleviate NMOSD-associated NP, in which median NRS pain scores decreased from 5.0 to 1.5 within 10 days.^[20]^

PRF, featuring simple operation, minimal invasiveness, safety, effectiveness, and minimal damage, is a novel nonpharmacological pain therapy that has recently been used in the clinical setting to treat various intractable NP.^[15,16,21,22]^ The exact analgesic mechanism of PRF remains unclear. At present, it is hypothesized that an electromagnetic field is generated around the target nerve, thus activating pain inhibitory pathways, reducing norepinephrine and 5-hydroxytryptamine,^[23]^ inhibiting the activity of excitatory nociceptive C-fibers,^[24,25]^ reducing the release of excitatory amino acids such as glutamate,^[26]^ inhibiting pro-inflammatory cytokines,^[27,28]^ activating dorsal horn pain-processing neurons,^[29]^ and reducing microglial activation.^[30]^ It is well known that PRF is effective in relieving peripheral NP, such as postherpetic neuralgia, carpal tunnel syndrome, occipital neuralgia, etc.^[24]^ However, the efficacy of PRF in central NP is being explored. Reports have suggested that PRF is effective in trigeminal neuralgia caused by multiple sclerosis^[31]^ and poststroke complex regional syndrome,^[32]^ and there is no report of PRF being used to treat NMOSD-associated NP. We suggest that, based on the success of this case, PRF is a nonpharmacological option for the treatment of central NP due to NMOSD and an alternative mechanism for pain management.

We performed the procedure under ultrasound guidance to avoid damage to the surrounding vital tissues and nerves. Ultrasound guidance allows real-time visualization of the puncture process, avoiding nerve damage from repeated punctures and damage to surrounding tissues without radiation. The procedure has been extensively used in the field of pain medicine.

In conclusion, we report the first case of NP caused by NMOSD successfully treated with PRF, suggesting that PRF could be helpful in the management of intractable NMOSD-associated NP. More studies are required to clarify the effect of PRF.

## Acknowledgments

The authors are grateful to the patient for providing permission to share the medical information.

## Author contributions

**Conceptualization:** Changxue Wu.

**Visualization:** Fei Yang, Liheng Lin, Yu Xia.

**Writing – original draft:** Fei Yang, Liheng Lin.

**Writing – review & editing:** Changxue Wu.
